# Pitfalls of practicing cancer epidemiology in resource-limited settings: the case of survival and loss to follow-up after a diagnosis of Kaposi’s sarcoma in five countries across sub-Saharan Africa

**DOI:** 10.1186/s12885-016-2080-0

**Published:** 2016-02-06

**Authors:** Esther Freeman, Aggrey Semeere, Megan Wenger, Mwebesa Bwana, F. Chite Asirwa, Naftali Busakhala, Emmanuel Oga, Elima Jedy-Agba, Vivian Kwaghe, Kenneth Iregbu, Antoine Jaquet, Francois Dabis, Habakkuk Azinyui Yumo, Jean Claude Dusingize, David Bangsberg, Kathryn Anastos, Sam Phiri, Julia Bohlius, Matthias Egger, Constantin Yiannoutsos, Kara Wools-Kaloustian, Jeffrey Martin

**Affiliations:** Department of Dermatology, Massachusetts General Hospital, Harvard Medical School, Bartlett Hall 6R, 55 Fruit Street, Boston, MA 02114 USA; Infectious Diseases Institute, Makerere University, Kampala, Uganda; University of California, San Francisco, USA; Mbarara University of Science and Technology, Mbarara, Uganda; Indiana University School of Medicine, Indianapolis, IN USA; AMPATH, Moi University, Eldoret, Kenya; Institute of Human Virology, Abuja, Nigeria; University of Abuja Teaching Hospital, Abuja, Nigeria; National Hospital of Abuja, Abuja, Nigeria; INSERM U897 & ISPED, Université Bordeaux, Bordeaux, France; R4D International, Yaounde, Cameroon; Regional Alliance for Sustainable Development, Kigali, Rwanda; Center for Global Health, Massachusetts General Hospital, Boston, MA USA; Albert Einstein College of Medicine/Montefiore Medical Center, Bronx, NY USA; Lighthouse Trust Clinic, Lilongwe, Malawi; University of Bern, Bern, Switzerland; Indiana University Fairbanks School of Public Health, Indianapolis, USA

**Keywords:** Survival, Mortality, Kaposi’s sarcoma, HIV/AIDS, Cancer, Resource-limited settings, Africa, Loss to follow-up, Cohort

## Abstract

**Background:**

Survival after diagnosis is a fundamental concern in cancer epidemiology. In resource-rich settings, ambient clinical databases, municipal data and cancer registries make survival estimation in real-world populations relatively straightforward. In resource-poor settings, given the deficiencies in a variety of health-related data systems, it is less clear how well we can determine cancer survival from ambient data.

**Methods:**

We addressed this issue in sub-Saharan Africa for Kaposi’s sarcoma (KS), a cancer for which incidence has exploded with the HIV epidemic but for which survival in the region may be changing with the recent advent of antiretroviral therapy (ART). From 33 primary care HIV Clinics in Kenya, Uganda, Malawi, Nigeria and Cameroon participating in the International Epidemiologic Databases to Evaluate AIDS (IeDEA) Consortia in 2009–2012, we identified 1328 adults with newly diagnosed KS. Patients were evaluated from KS diagnosis until death, transfer to another facility or database closure.

**Results:**

Nominally, 22 % of patients were estimated to be dead by 2 years, but this estimate was clouded by 45 % cumulative lost to follow-up with unknown vital status by 2 years. After adjustment for site and CD4 count, age <30 years and male sex were independently associated with becoming lost.

**Conclusions:**

In this community-based sample of patients diagnosed with KS in sub-Saharan Africa, almost half became lost to follow-up by 2 years. This precluded accurate estimation of survival. Until we either generally strengthen data systems or implement cancer-specific enhancements (e.g., tracking of the lost) in the region, insights from cancer epidemiology will be limited.

## Background

Survival after diagnosis is one of the most fundamental parameters in cancer epidemiology. Survival encompasses both the natural biologic history of a malignancy as well as the effects of therapeutic interventions. Monitoring survival over time, for example, can be a powerful tool to measure the cumulative impact of medical advancements. In resource-rich settings, ambient clinical systems diagnose cancers as they occur in the community, municipal registries record all deaths, and well-established cancer registries and epidemiologic platforms (such as the Surveillance, Epidemiology and End Results (SEER) program in the U.S. [1]) combine and synthesize data to make cancer survival estimation in real-world populations accurate and straightforward. In resource-poor settings, the importance of cancer has recently drawn attention [2–4], but, given the deficiencies in healthcare information systems in these regions, it is less clear how well we can determine cancer survival with ambient data.

Kaposi’s sarcoma (KS) in sub-Saharan Africa is an example of a malignancy in a resource-limited setting which would benefit from knowledge about current survival. KS was among the more common cancers in Africa even before HIV [5, 6], exploded in incidence in the HIV era [7–9], and exhibited poor survival early on in the HIV epidemic [8, 10]. More recently, antiretroviral therapy (ART) has substantially improved KS survival in resource-replete settings [11–13] and is now becoming more widely available in resource-poor settings [14]. However, the finding of improved survival in the ART era in resource-rich settings, such as the U.S., cannot automatically be extrapolated to resource-poor settings, such as Africa. Important differences in availability of oncologic care [15], co-morbidities [16], as well as differences in human host and viral etiologic pathogen [17] suggest that we must examine KS survival directly in sub-Saharan Africa [18–21] if we hope to understand the impact of the ART era.

To address whether ambient data can answer a fundamental question of cancer survival in sub-Saharan Africa, we examined the feasibility of estimating survival after a KS diagnosis in the current era of burgeoning ART use. We took advantage of an epidemiologic network of clinical data from throughout sub Saharan Africa [22] to identify a large community-representative sample of KS cases in five countries. We then combined these clinical data with all other available administrative data to attempt to estimate survival after KS diagnosis.

## Methods

### Design and study population

Among HIV-infected adults diagnosed with KS, we performed a cohort analysis of time to death using ambient clinical and administrative data. We retrospectively identified a consecutive sample of HIV-infected adults (≥18 years old) diagnosed with KS from January 2009 to July 2012 who were receiving their primary care at one of thirty-three HIV clinics in Kenya, Uganda, Malawi, Nigeria and Cameroon. The sites included 26 clinics in a network in western Kenya (Academic Model Providing Access to Healthcare (AMPATH)), one clinic in Uganda (Immune Suppression Syndrome Clinic (ISS) in Mbarara), two clinics in Nigeria (University of Abuja Teaching Hospital and National Hospital of Abuja), three clinics in Cameroon (Limbe Regional Hospital, General Hospital of Yaoundé and Military Hospital of Yaoundé), and one clinic from Malawi (Lighthouse Trust in Lilongwe). Each site administers ART in accordance with their national guidelines, and each participates in the International Epidemiologic Databases to Evaluate AIDS (IeDEA) Consortium, which, in Africa, consists of East, West, Central and Southern Africa regions [22]. IeDEA was established in 2005 to harmonize diverse HIV/AIDS-related data collected as part of routine clinical care in seven regions throughout the world [23]. All patients provided written consent for data derived from their care at the participating clinic sites to be used for purposes of research through IeDEA. No children were involved in this study. Ethics committees that approved this study are listed in the Funding section of this paper.

### Measurements

At the participating clinics, KS diagnosis was made during the course of routine clinical care, either by clinical examination alone or with biopsy confirmation. Both individuals who were diagnosed at their initial clinic visit and during the course of their care were included. Ascertainment of death was through review of clinic charts, clinic databases and municipal death registries, where available. Clinics are informed of a patient’s death in a variety of ways, including from reports from adjacent hospital units or from family members. All of the participating clinics had some form of a tracking program in place to follow-up on lost patients. Demographic characteristics, including age and sex, were routinely collected by the clinics and extracted from the respective clinic databases. CD4+ T cell counts were performed at clinical laboratories associated with each of the clinics. The CD4 count proximal to KS diagnosis, when available, was defined as CD4 count closest to date of diagnosis, within 180 days prior to KS diagnosis or up to 14 days after.

### Statistical analysis

Time zero for the analysis was date of KS diagnosis. Patients were followed until death, and for those not known to be dead, the last known date known to be alive either from a clinic visit or transfer to another facility. Cumulative survival following KS diagnosis was estimated via the Kaplan-Meier technique, with censoring at transfer or last clinic visit. This approach assumes that the rate of death among those lost to follow-up is the same as those whose disposition is known. Loss to follow-up was defined as absence from clinic during the 3 months prior to database closure with no evidence of death or transfer. Incidence of loss to follow-up was calculated using the Aalen-Johansen estimator with death as a competing event [24, 25]. Proportional hazards regression was used to evaluate the independent association between various exposures measured at the time of KS diagnosis (age, sex and proximal CD4 count) and loss to follow-up. For the regression analysis, missing exposure variables were accommodated for with multiple imputation, with ten copies, performed with the “mi impute” command in Stata version 12.1 (Stata Corp., College Station, Texas) [26, 27]. Standard errors were calculated using Rubin’s rules, which account for the variability in results between the imputed datasets [27].

## Results

Across the 33 HIV clinics, we analyzed data from 1328 adults diagnosed with KS during the course of primary care for HIV disease in all four African regions in the IeDEA Consortium. There were 677 cases from Kenya, 172 from Uganda, 57 from Nigeria, 67 from Cameroon and 355 from Malawi. Overall, 40 % were women, the median age was 35 years (interquartile range (IQR): 30–41), and the median proximal CD4 + T cell count was 159/mm^3^ (IQR: 59–299) at time of KS diagnosis (Table 1). Most of the KS diagnoses (72 %) were made on clinical grounds alone without biopsy.

**Table 1:** Characteristics of HIV-infected patients diagnosed with Kaposi’s sarcoma between 2009–2012 in Kenya, Uganda, Nigeria, Cameroon and Malawi

	AMPATH Kenya (*n* = 677)	ISS Uganda (*n* = 172)	UATH and NHA Nigeria (*n* = 57)	Yaounde and Limbe Cameroon (*n* = 67)	Lighthouse Malawi (*n* = 355)	Total (*n* = 1328)
Male sex, %^a^	60 %	60 %	49 %	49 %	72 %	60 %
Age, years^a^	35 (30–42)^b^	33 (28–40)	36 (30–41)	35 (30–41)	34 (30–40)	35 (30–41)
CD4+ T cell count/μl^c^						
≤ 50	25 %	24 %	33 %	17 %	11 %	23 %
51-200	32 %	31 %	17 %	50 %	46 %	35 %
201-350	21 %	28 %	39 %	21 %	30 %	24 %
> 350	21 %	16 %	11 %	12 %	13 %	18 %

*AMPATH* denotes Academic Model Providing Access to Healthcare, *ISS* denotes Immune Suppression Syndrome Clinic, *UATH* denotes University of Abuja Teaching Hospital, *NHA* denotes National Hospital of Abuja

^a^two missing values for sex, four missing values for age

^b^median (interquartile range)

^c^CD4+ T cell count proximal to KS diagnosis, defined as closest CD4 count to date of KS diagnosis within the period 180 days prior to diagnosis to 14 days after diagnosis. CD4 data is missing in 33 % of patients. Data presented represent observed (not imputed) values only

**Fig. 1:**
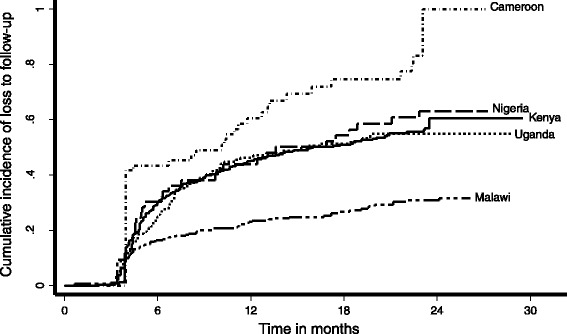
Cumulative incidence of loss to follow-up in HIV-infected patients following diagnosis with Kaposi’s sarcoma in five countries in sub-Saharan Africa

Patients were followed for a median of 8.9 months (IQR: 4.0–20) and a collective 1473 person-years in which 191 patients died. Cumulative mortality, as estimated by the Kaplan-Meier technique, was 13 % at 6 months following KS diagnosis, 18 % at 1 year, and 22 % at 2 years. These estimates of mortality were clouded, however, by a cumulative loss to follow-up of 23 % (95 % confidence interval (CI): 21–26 %) at 6 months, 36 % (95 % CI: 33–38 %) at 1 year, and 45 % (95 % CI: 42–48 %) at 2 years (Fig. 1). This cumulative loss to follow-up takes death into account as a competing event. At no site was there an accessible municipal death registry or any other administrative source in which to search for deaths among those deemed to be lost.

Given the high frequency of lost to follow-up, we also explored its determinants. After adjustment for geographic site in proportional hazards regression, age <30 years (hazard ratio (HR) 1.41, 95 % CI: 1.11–1.79) and male sex (HR 1.35, 95 % CI: 1.12–1.63) were independently associated with loss to follow-up (Table 2). Those with CD4 count ≤50 cells/mm^3^ also had a higher incidence of becoming lost (HR 1.23, 95 % CI: 0.90–1.67, *p* = 0.19), although this did not meet conventional levels of statistical significance. When the analysis was restricted to those with biopsy-proven KS (*n* = 378), the results were largely unchanged, with the exception that CD4 count ≤50 cells/mm^3^ was significantly associated with loss to follow-up (HR 2.35, 95 % CI: 1.29–4.27, *p* < 0.01).

**Table 2:** Unadjusted and adjusted proportional hazards regression evaluating factors associated with loss to follow-up among HIV-infected patients with Kaposi’s sarcoma from Kenya, Uganda, Nigeria, Cameroon and Malawi

Characteristic	Unadjusted		Adjusted^a^	
	Hazard Ratio (95 % CI)	*P* value	Hazard Ratio (95 % CI)	*P* value
Age, years				
≥ 40	Reference		Reference	
35–39	1.00 (0.78–1.29)	0.96	1.06 (0.82–1.36)	0.69
30–34	1.01 (0.80–1.28)	0.93	1.09 (0.85–1.38)	0.50
< 30	1.30 (1.03–1.64)	0.03	1.41 (1.11–1.79)	0.005
Sex				
Female	Reference		Reference	
Male	1.08 (0.91–1.30)	0.38	1.35 (1.12–1.63)	0.002
CD4+ T cells, count/μl				
> 350	Reference		Reference	
201–350	1.00 (0.74–1.35)	0.99	1.03 (0.75–1.42)	0.84
51–200	1.10 (0.82–1.49)	0.52	1.14 (0.84–1.54)	0.24
≤ 50	1.25 (0.92–1.68)	0.15	1.23 (0.90–1.67)	0.19

^a^adjusted for geographic clinic site (country), age, sex and CD4+ T cell count

## Discussion

When attempting to estimate survival in a large HIV primary care-based sample of patients diagnosed with KS spanning five countries in sub-Saharan Africa, we found that almost half were lost to follow-up by the end of 2 years. Because the fraction of those lost is so large and the disposition of the lost is unknown, it was therefore not possible to estimate with any certainty a fundamental parameter in cancer epidemiology — survival — using data from available clinical and administrative systems. As noted earlier, for a technique like the Kaplan-Meier method to yield an estimate of survival in the face of loss to follow-up, it requires an assumption that those who are lost to follow-up have the same outcomes as those who remain under observation. Since this “non-informative censoring” assumption is likely implausible in our context given that a higher portion of the lost patients may in fact have died [28], our Kaplan-Meier estimate of survival is essentially uninterpretable.

The substantial loss to follow-up that we observed is consistent with findings from other cancers in sub-Saharan Africa [29, 30], as well as KS specifically. Although the methodologic approaches and metrics vary, reported loss to follow-up for KS at 1 year or less after diagnosis in Africa has ranged from 15 to 23 % [18, 19, 21] and was 26 % at 2 years in another report [20]. Our work, which found an even higher incidence of lost to follow-up, bolsters these earlier findings in several ways. First, the IeDEA consortium is a large and diverse population, which for this project included patients from East, West, Central and Southern Africa, thus enhancing the generalizability of the findings. Second, the patients were not selected from oncology clinics, clinical trials, registries or a tightly monitored research cohort. Instead, they were — by design — identified at the time of their initial diagnosis in real-world community-based HIV primary care settings and their subsequent observation was entirely without research influence. Indeed, because we identified patients directly from HIV primary care clinics, irrespective of whether they had a confirmatory biopsy, our population is likely different than many registry-based cancer populations which primarily identify KS diagnoses from pathology laboratories. Since we do not know which types of patients with KS in Africa obtain a biopsy diagnosis (e.g., they may have more severe disease, or higher socioeconomic status), it is unclear if patients who receive biopsies are representative of the larger population of all incident KS. Therefore, if the target is to encompass all new KS diagnoses in Africa, we believe that our estimate of the incidence of loss to follow-up among persons with KS is among the least biased to date.

We were unable to determine survival due to the high loss to follow-up and the unknown disposition of those that are lost to follow-up. In prior work from East Africa that assessed a consecutive sample of HIV-infected adults attending HIV clinics (including two clinics participating in this project), we actively sought after those who were lost by searching for them in the community [31]. We found that three possible outcomes occur in considerable proportions: death, previously undocumented transfer to another facility, or alive but discontinued care [32, 33]. Specifically, in this prior work, the cumulative incidence of mortality at 1 year among those that were lost was ultimately found to be 36 %, once the patients had been tracked [32, 33]. Such vital status estimates, derived from a general HIV cohort, are useful but cannot be relied upon to accurately estimate mortality rates in selected sub-populations such as patients with KS. Likewise, it is not likely that nomogram approaches to correct survival in the face of lost to follow-up that were developed for all HIV-infected patients on ART (irrespective of KS) [34] will perform adequately amongst patients with KS. Work from South Africa demonstrates that loss to follow-up is higher in KS patients compared to other HIV-infected patients starting on ART [21]. We speculate that this is because a larger fraction of patients with KS die, their deaths go unrecognized by their primary care clinics, and, hence, they are deemed lost to follow-up. Indeed, the higher incidence of loss among those with CD4 ≤ 50 cells/mm^3^, while not statistically significant unless restricted to those with biopsy-proven diagnosis, does suggest that those who were lost became lost because of death. Therefore, the nominal estimate of survival we observed using available data is likely a substantial overestimate. Without ascertaining the outcomes of those who are lost, we will never understand true survival after a diagnosis of KS in sub-Saharan Africa.

A limitation of this work is that many of the KS diagnoses are based on clinical suspicion only. The high frequency of clinical diagnosis of KS has been documented by others [35] and is largely due to the limited biopsy infrastructure in sub-Saharan Africa [36]. Work from East Africa has shown that there are many conditions that can clinically mimic KS [37]; it is possible that our study population, therefore, includes patients with conditions other than KS. Because we suspect that many more clinical mimickers of KS have more favorable (as opposed to less favorable) prognosis compared to true KS, we again believe that our nominal estimate of survival is an overestimate of truth. In addition, due to atypically rigorous tracking of the lost at one of our sites (Lighthouse Clinic in Malawi), we may actually underestimate the proportion of lost as it compares to a general African clinic population. Finally, although not a threat to the internal validity of the overall findings, the sites contributed sizably different numbers of KS cases, which is in a large part a reflection of underlying differences in KS epidemiology across sub-Saharan Africa.

## Conclusions

In summary, this work demonstrates on a large scale the challenges of accurately estimating cancer survival in sub-Saharan Africa. This issue is gaining significance as interventions (such as chemotherapy for KS) become more readily available, such that monitoring survival over time is increasingly important. Until we either generally strengthen data systems or implement cancer-specific enhancements to derive more accurate survival estimates (e.g., tracking of the lost patients with cancer in the community) in the region, insights from cancer epidemiology will be severely limited. Strengthening data systems across this entire region may not be possible in the short term, but sentinel regional sites could be selected for enhanced monitoring and tracking of the lost. Additionally, the recent expanding efforts in cancer registries in sub-Saharan Africa [38–40] will need to closely address the issue of loss to follow-up in order to truly provide added value.

## Abbreviations

AIDS: acquired immune deficiency syndrome
AMPATH: Academic Model For Prevention and Treatment of HIV
ART: antiretroviral therapy
HIV: human immunodeficiency virus
IeDEA: International Epidemiologic Databases to Evaluate AIDS
ISS: Immune Suppression Syndrome Clinic
KS: Kaposi’s sarcoma
SEER: Surveillance, Epidemiology and End Results
